# Picture quality between DSLR cameras and smartphone cameras among prosthodontics postgraduate students

**DOI:** 10.6026/973206300212226

**Published:** 2025-07-31

**Authors:** Varsha Mangtani, Anuja Tripathi, Alka Gupta, Mukesh Soni, Harsh Chansoria, Shivani Gupta

**Affiliations:** 1 Department of Prosthodontics and Crown & Bridge, Government College of Dentistry, Indore, Madhya Pradesh, India; 2 Department of Orthodontics and Dentofacial Orthopaedics, Sri Dharmasthala Manjunatheshwara College of Dental Sciences, Dharwad, Karnataka, India

**Keywords:** Digital single-lens reflex (DSLR) cameras, dental photography, image quality, post-graduate students, smartphone cameras

## Abstract

The line between smartphone and Digital single-lens reflex (DSLR) cameras is becoming less clear, especially in dental photography. This study assessed the knowledge of prosthodontic postgraduate students regarding image quality from both camera types. Approximately 300 students participated in a questionnaire that covered their knowledge, practices and opinions on DSLR versus smartphone photography. Data were analyzed using simple percentages. The results showed that DSLR cameras are still preferred for dental photography among these students.

## Background:

Clinical photographic images are invaluable tools in dental treatment, serving multiple purposes. They facilitate patient documentation, aid in communication between dental professionals and laboratory technicians, enhance patient understanding, support marketing efforts and enable the evaluation of treatment outcomes [1]. Additionally, they play a crucial role in identifying changes in facial patterns, which are particularly important in prosthodontics, oral surgery and oral rehabilitation. More recently, these images have found significant application in digital smile design [2]. Several dental camera models are available, ranging from simple compact devices to sophisticated, robust systems. A fundamental understanding of photography principles, including light control, aperture, shutter speed and sensor sensitivity, is essential for effective clinical photography [3]. Compact models, such as smartphones, offer user-friendly operation and integrated camera and lens units. These devices typically have limited adjustable features and fixed focal lengths, usually ranging from 29 to 33 mm. Lower focal lengths are ideal for capturing wide-angle panoramic views due to their broader field of view [4]. A Digital Single-Lens Reflex (DSLR) camera is essential for capturing high-quality images of both extraoral and intraoral structures. DSLR cameras offer superior durability, a wide range of adjustable settings and the flexibility to interchange lenses, allowing for various photographic needs.

The camera body houses the viewfinder, shutter release button, image sensor, shutter mechanism and often includes a built-in flash [5]. The lens, which is responsible for transmitting the image to the camera sensor, plays a crucial role in determining image quality. Macro lenses are particularly well-suited for dental photography, as they enable precise focusing and high-quality imaging of small objects. A DSLR camera equipped with a high-quality macro lens is considered the ideal choice for dental photography, as it allows for capturing both portraits and close-up or macro images of the dentition through the lens viewfinder. This configuration ensures accurate focusing, framing and exposure metering [6]. Given the widespread accessibility, affordability and significant advancements in smartphone camera technology, they have become a viable option for capturing dental images in many situations. Smartphone cameras have undergone substantial improvements, with resolutions increasing from less than 1 megapixel to over 20 megapixels. Additionally, the transition from CCD sensors to CMOS sensors has enhanced image quality, accuracy and sensitivity [7]. A comparison of images obtained with different devices should help dentists choose the most suitable equipment. The primary factor contributing to the widespread adoption of smartphones as primary cameras is their ubiquitous nature as essential tools for daily life. This ensures that most individuals always have a high-quality camera with them, aligning with the adage popularized by iPhone icon Chase Jarvis: "The best camera is the one you have with you." Therefore, in this study, we aimed to compare the image quality of smartphone photographs with that of DSLR images, considered the gold standard in photography.

## Methodology:

## Study setting and design:

This study was designed as an observational, cross-sectional, web-based questionnaire survey. Ethical approval was obtained from the Institutional Review Board prior to commencement. A convenience sampling method was employed, inviting postgraduate students in Prosthodontics from various dental institutes across Central India to participate voluntarily in the survey.

## Questionnaire development:

The research questionnaire was developed based on existing literature and previous studies. It comprised 25 structured questions divided into five sections: demographic information, preferred mode of dental photography, key attributes influencing image quality, light sources used to enhance image quality, lens and resolution preferences and software utilization. The questionnaire included a mixture of open-ended, closed-ended, yes/no, multiple-choice and contingency questions. The questionnaire was administered using Google Forms, with a survey link distributed via email and WhatsApp to selected dental institution groups. The survey format included a participant information sheet, an online informed consent form, the questionnaire and a closing thank-you note. An open-ended question was included at the end to collect participants' suggestions. To ensure data integrity, responses were limited to one per participant. The participant information sheet outlined details about the primary investigators and the study objectives. Participation was entirely voluntary and responses were automatically recorded in a linked spreadsheet.

## Participants and data collection:

The survey was distributed to a total of 300 postgraduate students in Prosthodontics from multiple dental institutions across Central India. Data collection spanned one month.

## Statistical analysis:

Collected data were compiled and analyzed using IBM SPSS Statistics version 21.0. Descriptive statistics were presented as frequencies and percentages. The Chi-square test was employed to evaluate the association between postgraduate students' academic year and their knowledge, attitudes and perceived barriers related to digital learning via smartphones. Statistical significance was set at P < 0.05.

## Results and Discussion:

Figure 1 (see PDF) depicts preferred dental photography modes among MDS students, revealing distinct patterns across academic years and genders. Digital camera use was exclusively reported by first-year students, with females comprising the entire group in this category. Smartphone usage was predominant overall, particularly among second-year students, where usage was nearly balanced between males and females. In the third year, smartphone preference remained notable, but it skewed more toward male students, whereas in the first year, female students led in smartphone use. The combined use of both digital cameras and smartphones was relatively low, mainly observed in second-year male students and a smaller proportion of first-year female students, with no representation from third-year students. These findings suggest a shift toward smartphone reliance as students advance in their studies, alongside clear gender differences in device preferences for dental photography. Among MDS students, smartphone usage varied notably across academic years. In the first year, all smartphone users exclusively used iOS devices. The second year showed a more diverse pattern, with students nearly evenly split between Android and iOS users, along with a significant portion who did not use smartphones at all. By the third year, a majority of students reported not using any smartphones, while the remainder primarily used Android devices, with fewer students using iOS devices. This progression reflects a shift from exclusive iOS preference in early study years toward increased variability and reduced smartphone usage in later years. Digital camera usage among MDS students varied across academic years and brands. In the first year, all digital camera users exclusively used Canon cameras. The second-year students displayed a more varied distribution, with Nikon being the most used brand, followed by Sony, Canon and a notable portion of students not using any digital camera. In the third year, Sony cameras became the predominant choice, with Canon also used by a substantial number of students, while Nikon usage dropped significantly. Additionally, a smaller segment of third-year students reported not using any digital camera at all. This trend suggests a shift in brand preferences and a diversification of digital camera usage as students' progress through their studies. Image quality attributes preferences among MDS students varied distinctly by academic year. In the first year, brightness was unanimously rated as the most important attribute. Second-year students prioritized sharpness, with more than half highlighting its importance, followed by brightness and contrast. In the third year, colourfulness emerged as the leading attribute of importance, with nearly half the students selecting it, while brightness and contrast were less frequently emphasized. This progression reflects evolving perceptions of key image quality attributes as students advance in their studies (Figure 2 - see PDF).

The data on smartphone image quality perceptions among MDS students indicate differences by academic year and gender. Female students in the second year most frequently acknowledged the importance of smartphone image quality, with 62 reporting a positive response, followed by first-year females (47 in number) and third-year females (28 in number). Male students showed a less consistent pattern, with 40 in the first year and 37 in the third year reporting a negative response regarding the importance of smartphone image quality and no males in the second or third year reporting it as important. Overall, female students demonstrated a higher recognition of the significance of image quality in smartphones compared to male students, particularly in the early and middle years of the program. The data on lighting preferences among MDS students show clear trends in the types of light used for dental photography. Natural light was the most preferred lighting condition across all years, especially among female students, with 54 first-years, 72 second-year and 35 third-year females favoring this option. Male students also predominantly preferred natural light, with 22, 27 and 37 males in the first, second and third years, respectively, choosing it. Fluorescent light was used exclusively by female students in the second year (9 in number) and first year (12 in numbers). Incandescent light was chosen only by first-year male students (25). A small number of female students in the first year (7 in number) reported using other types of light. This data suggests a strong overall preference for natural light in dental photography, with minor variations in the use of alternative light sources based on gender and academic year (Figure 3 - see PDF). The data on lighting preferences for dental photography among MDS students indicate a strong preference for natural light across all academic years and genders. Natural light was favored particularly by female students in the second year (72 in number), followed by first-year females (54 in number) and third-year females (35 in number). Male students also preferred natural light, with 37 in the third year, 27 in the second year and 22 in the first year. Fluorescent light was chosen exclusively by female students, with 9 in the first year and 12 in the second year opting for it. Incandescent light was selected by only male students in the first year (25 in number). Additionally, a small number of female students (7 in number) in the first year reported using other types of light sources. These findings highlight the predominant use of natural light in dental photography, with some variations in the use of alternative light sources based on gender and academic year (Figure 4 - see PDF).

The data on lens usage for image quality among MDS students reveal that a majority do not use specialized lenses. Among female students, 77 in the second year and 61 in the first year reported not using lenses, while 16 second-year and two third-year females indicated lens use. Among males, lens usage was notably lower, with only 25 first-year males reporting using lenses, while 37 third-year and 27 second-year males reported no lens use. Regarding the importance of specific image resolution, the majority of female students in the first (54) and second (43) years reported not emphasizing specific resolution, while 50 second-year females considered it important. Male students predominantly did not prioritize particular image resolution, with 47 first-year males and 27 second-year males responding negatively and no males reported considering it important. This data highlights a general tendency not to use specialized lenses or focus on specific image resolution, with some variation across gender and academic years. The data regarding the use of editing applications among MDS students indicate gender and year-wise variations. For digital camera editing apps, a majority of female second-year students (65 in number) reported not using any editing applications. In contrast, 27 first-year and 28 second-year females did use such apps. Among male students, 47 first-year and 37 third-year students did not use editing apps, with a smaller number of males (13 in number) in the second year using them. Regarding smartphone editing apps, female students in the second year again showed the highest proportion not using editing apps (84 in number), whereas 17 first-year and 14 third-year females reported using them. Male students showed a more balanced distribution, with 35 first-year and 25 third-year males not using editing apps and 12 males each in the second and third years reporting usage. This data highlights a trend of lower editing app usage among females, particularly in the second year and a generally moderate engagement with editing apps across genders and academic years. Regarding perceptions of better image quality among MDS students, digital cameras were predominantly favored by females across all years, with 73 second-year, 61 first-years and 35 third-year females indicating this preference. Males showed a moderate preference for digital cameras, especially in the first year (22 in number) and second year (20 in numbers). Android smartphones were also considered to provide better image quality by some females (13 second-year students) and males (25 first- and third-year students), while iOS devices were less frequently cited. In prosthodontics-related photography, females mainly used digital cameras in the first (61 in number) and second years (57 in numbers) and to a lesser extent by males in the first (22 in number) and third years (20 in numbers). Smartphone cameras for prosthodontics were predominantly used by second-year females (36 in number) and third-year males (37), with lower usage among first-year males and females. These findings illustrate a preference for digital cameras for better image quality and prosthodontic photography, with smartphones playing a significant role in the mid-to-late years, particularly among males.

The present study corroborates earlier research emphasising the critical role of high-quality photographic imaging in dental practice, particularly within prosthodontics. Consistent with prior findings, DSLR cameras continue to be regarded as the gold standard for intraoral photography due to their superior image quality, versatility and specialized features such as macro lenses, which facilitate optimal focal length and magnification necessary for capturing fine oral structures with minimal distortion. These attributes align with established evidence highlighting the advantage of DSLR systems in providing precise exposure control and consistent framing, essential factors for accurate clinical documentation and treatment planning as discussed by Desai *et al.* (2013) [8]. Parallel to this, the study reflects the evolving role of smartphone cameras, which have rapidly advanced in technological capabilities and ease of use. Echoing recent literature, smartphones offer significant practical benefits, including portability, immediacy and user-friendliness, enabling even non-specialists to produce clinically acceptable images despite inherent limitations such as shorter focal lengths and potential distortion in close-range captures. This dual recognition aligns with contemporary studies suggesting that while smartphones cannot fully replace DSLRs in clinical precision, they provide an accessible and efficient alternative for routine documentation and initial assessments as discussed by Patonis *et al.* 2024 [9]. In terms of image quality, the study by Prasad AS *et al.* [10] and Patussi *et al.* [11] found that DSLR cameras provide sharper, more detailed images with better depth of field, which is essential for capturing intricate oral structures and complex orthodontic cases. DSLR cameras also offer greater flexibility in terms of lens options, allowing for a more precise control over focal length and magnification, which is important in orthodontics for capturing accurate representations of malocclusions, tooth alignment, and other subtle features. Moreover, this study highlights the multifactorial determinants of image quality, including operator skill, lighting conditions, framing, focus and post-processing, which aligns with comprehensive frameworks proposed in previous research. By incorporating prosthodontists' perceptions, the present findings add to the growing evidence base advocating a balanced approach that weighs technical superiority against clinical practicality, ensuring optimal photographic outcomes tailored to varied clinical contexts.

## Conclusion:

Evaluating dental photograph quality is vital for improving oral healthcare. This study shows that both DSLR and smartphone cameras produce high-quality images, with smartphones offering convenience and efficiency. However, DSLR cameras are preferred by postgraduate prosthodontic students for capturing finer details in clinical documentation.
